# Patient Expectation in China: Exploring Patient Satisfaction in Online and Offline Patient–Provider Communication

**DOI:** 10.3389/fpsyg.2022.888657

**Published:** 2022-06-10

**Authors:** Bolin Cao, Dongya Wang, Yifan Wang, Brian J. Hall

**Affiliations:** ^1^School of Media and Communication, Shenzhen University, Shenzhen, China; ^2^Center for Global Health Equity, New York University Shanghai, Shanghai, China; ^3^Bloomberg School of Public Health, Johns Hopkins University, Baltimore, MD, United States

**Keywords:** online patient–provider communication, patient satisfaction, online medical consultation, eHealth, doctor–parent communication

## Abstract

**Introduction:**

Online patient–provider communication (OPPC) has become an alternative approach to seek medical advice and contact health professionals. However, its penetration rate remains low, and the underlying mechanisms of patient satisfaction with OPPC are underexamined. This study investigates the role of patient expectancy and the expectancy violation of patient-centered communication (PCC) in patient satisfaction in emerging OPPC scenarios by integrating the concepts of PCC and expectancy violation theory (EVT).

**Method:**

An online survey was conducted in October 2019 among Chinese respondents who experienced OPPC and offline medical services.

**Results:**

The 471 qualified participants reported high satisfaction with OPPC (mean [M] = 3.63, standard deviation [SD] = 0.81). However, patient satisfaction with OPPC was lower than that in offline medical encounters (*M* = 3.75, SD = 0.80), and patients suffered a higher expectancy violation of PCC in OPPC scenarios (*M* = 0.45, SD = 0.76) than in offline medical encounters (*M* = 0.27, SD = 0.69). Nevertheless, patients’ satisfaction with OPPC significantly increased as the frequency of OPPC usage increased (*β* = 0.209, *p* < 0.001). This positive relationship was partially mediated by the decrease in the expectancy violation of PCC in OPPC scenarios.

**Discussion:**

The study can contribute to increasing the adoption of OPPC and reducing the burden of offline medical resources.

## Introduction

The digital transformation of healthcare services brings us into an era where offline and online patient–provider communication (OPPC) coexist as part of our daily life (Agarwal et al., 2010). OPPC allows patients access to physician advice for their symptoms or stay connected with physicians by using internet technology-based applications (apps; Santana et al., 2010; Jiang, 2019a). Traditional offline medical visits remain the dominant format for healthcare provision, whereas OPPC is an alternative and supplementary approach to attenuate various problems relevant to healthcare (Gunter, 2005; Jiang et al., 2019).

Many medical apps and platforms have been developed under the encouragement and support of government policies, market demand, and consumer need (Shakhovska et al., 2019). These services have attracted an increasing number of users worldwide who log on to access online medical services (Santana et al., 2010; Silver, 2015; Jiang, 2019a). More than 561 apps can be found in the category “online doctors and telemedicine” in Android and iPhone app stores (Appgroves, 2020). The number of OPPC users remarkably increased, especially during the COVID-19 outbreak when social distancing was encouraged. The market share of OPPC is foreseen to grow with an estimated annual growth rate of 17.7% in 2020 (Med. China, 2020).

However, the development of OPPC remains in its infancy compared with many other online services (Jiang, 2019b). Some studies have been conducted to understand the feasibility, effectiveness, and user experience of OPPC, but these findings remain limited (Lee and Zuercher, 2017). Particularly, the mechanisms of patient satisfaction toward OPPC are underexplored. Patient satisfaction with OPPC is the driving force that fosters user habits and expands market share, which can further lead to better medical outcomes with OPPC (Hawthorne et al., 2014). Focusing on improving patient satisfaction with OPPC will lead to the increased uptake and adoption of OPPC.

In addition, patient-centered communication (PCC), which focuses on patients’ need, value, and preference, is vital to improve patient satisfaction and healthcare delivery (Jiang, 2017). PCC is promoted as a regular part of healthcare providers’ routine to ensure that they are meeting patient needs and are ensuring patient engagement throughout the medical process (Committee on Quality of Health Care in America, 2001). From the patient perspective, expectation and expectation violations of PCC can remarkably influence their experience and future usage of OPCC. Therefore, examining patient perception of PCC is highly remarkable for novel services, such as OPPC (Stewart et al., 2000; Ishikawa et al., 2013).

The purpose of this study is two-fold. First, this study aims to examine the effectiveness of OPPC through comparisons between online and offline patient–physician interaction. Second, this study investigates the roles of the perception of PCC (expectancy and expectancy violations) as a mediator in the relationship between OPPC and patient satisfaction.

## Literature Review

### Online Patient-Provider Communication

The widespread adoption of internet communication technologies has changed traditional healthcare in tremendous ways. Patient–provider communication (PPC) has also developed its digital format as a central part of healthcare service (Gunter, 2005). OPPC is a new term used to reflect the electronic communication between patients and providers throughout the medical process (Ye et al., 2010). Electronic communication channels include email, instant messages, social media, medical apps, and video conferences (Jiang et al., 2019). In the present study, the typical approach for OPPC was to use medical apps or social media for medical consultation.

The development of OPPC is at its early stage, but its potential has already been widely recognized (Gunter, 2005). First, OPPC is beneficial for various instrumental aspects. OPPC allows users to transcend physical barriers in time and space and cut patients’ cost on traffic and other costs for medical leaves (Lee and Zuercher, 2017). OPPC can also save patients’ waiting time and reduce access burden related to navigating the healthcare system (Baker et al., 2005). Second, the text-based nature of OPPC has advantages of documented consultation process and provision of a clear explanation of symptoms and instructions (Lee and Zuercher, 2017). Third, due to discrimination toward stigmatized diseases, including HIV and mental disorders, OPPC provides opportunities to maintain anonymity by creating a space where patients feel comfortable (Dunn, 2012). Fourth, OPPC can be an ideal approach for social support (Silver, 2015). The quick response received from OPPC can eradicate feelings of extreme anxiety. A meta-analysis found that most patients appreciate OPPC when they seek social support, especially in a resource-constrained environment (Wagg et al., 2018).

Although researchers recognized the increasing trend of OPPC development, some held less optimistic opinions on its adoption and influence. The principal concern is its low adoption rate at the current stage (Jiang, 2019a). Only 5.8% of the American population has used online means to contact their physicians, and less than 10% of the European population has communicated with healthcare providers online (Santana et al., 2010; Ye et al., 2010). Its low adoption rate has several reasons, and the major one is that people hold conservative opinions toward OPPC and are dubious about the effectiveness of OPPC without face-to-face consultation (Jiang, 2019b). Furthermore, changing the habits on medical service usage is difficult. Many people still visit physicians in person, and OPPC remains far from becoming a habit or a main choice for medical services (Gunter, 2005; Jiang, 2019b).

As a novel service, some barriers of OPPC await time to cross, but some walls can be overcome by better understanding the effectiveness and mechanisms of OPPC. OPPC has been linked to various positive outcomes, including enhancing patient adherence (Silver, 2015), fostering self-management (Hou and Shim, 2010), empowering patient decision-making (Fox et al., 2005), and obtaining positive physical and mental health outcomes (Jiang, 2019a). Nevertheless, patient satisfaction and consultation quality online are essential to further maximize its positive effects and foster a habit of using OPPC (Gunter, 2005; Jiang, 2019b). Therefore, we discuss the potential factors influencing patient satisfaction in OPPC.

### Patient Satisfaction and PCC

Patient satisfaction is an important indicator in evaluating medical treatment process and predicting medical outcomes (Hawthorne et al., 2014). Examining PCC can subsequently influence an individual’s healthcare utilization and broadly impact the overall progress of the OPPC domain (Stewart et al., 2000). Patient satisfaction refers to individuals’ subjective perceptions of the extent to which expected healthcare service is received during the medical service process (Agha et al., 2009). Numerous studies proposed a direct relationship between medical encounter behaviors and treatment outcomes; however, a growing number of analyses have uncovered that, rather than being directly associated, the relationship is mediated by patient satisfaction with their communication to healthcare providers (Street et al., 2009; Jiang, 2019a).

Patient satisfaction to medical services can be multifold and varies from the core effectiveness of treatment or medicine to the satisfaction of access to parking facilities surrounding the hospital (Altin and Stock, 2016). We specifically focus on patient satisfaction toward the communicative process with physicians during their medical consultation and treatment. Patient satisfaction, as a result of medical encounter behaviors, can also be a cause of medication adherence and treatment effects. Therefore, boosting patient satisfaction in the PPC process is considered highly conducive and prioritized.

PCC has been identified as a promising approach to increase patient satisfaction (Street et al., 2009). PCC emphasizes that healthcare should meet patients’ individual preferences, needs, and values for patients to obtain high-quality medical care. More specifically, Street (2003) conceptualized PCC as taking patients’ perspectives, understanding patients’ psychosocial context, and building shared understanding, power, and responsibilities throughout the healthcare process. Therefore, PCC reflects the benign communication between healthcare providers and patients and calls for patient involvement in medical care processes (Epstein et al., 2005).

The positive relationship between PCC and patient satisfaction has been documented. For example, a meta-analysis of 25 studies found that increased PCC is substantially associated with increased patient satisfaction in the context of cancer medical care (Venetis et al., 2009). The frequency of patients asserting treatment preferences, providers giving sufficient news, immediacy, and perceived listening are the PCC behaviors that are considerably associated with increased satisfaction level (Wanzer et al., 2004). In addition, underlying mechanisms exist between PCC and patient satisfaction (Jiang, 2020). These studies showcased that PCC empowers patients throughout the medical care process by enabling patients to perceive more autonomy and competency, which induce satisfaction and positive health outcomes (Venetis et al., 2009; Jiang, 2017). Thus, PCC is highly influential in patients’ satisfaction in the medical context.

### Expectancy and Expectancy Violation of PCC

Expectation is another important intervening factor that cannot be avoided in obtaining patient satisfaction (Moyer and Katz, 2007; Santana et al., 2010). Whether the expectation of PCC is satisfied or violated may highly influence patient satisfaction in terms of communicating with a physician (Dean et al., 2019). A closely relevant theory that tackles and assumes that individuals have expectancy toward others’ behaviors is the EVT. This theory was first coined by Burgoon and Jones (1976) to explain how individuals interpret and evaluate perceived proxemic violations caused by others. Nowadays, EVT has been widely used to study the impact of violation on interactions and/or relationships (Afifi and Metts, 1998). The central construct of EVT is expectancy. Slightly different from expectation, expectancy is a consistent pattern of anticipated behaviors that vary in different contexts and/or relationships among individuals (Burgoon, 1993).

Expectancy can be a double-edged sword. Experiences that meet or exceed expectancy can receive high satisfaction and greater behavior change in the direction advocated by the message. However, expectancy violation can potentially lead to low satisfaction and no or negative behavior change. In theory, expectancy violation can be positive or negative (Burgoon and Le Poire, 1993). The valence and perceived importance of a violation are also significant influencing factors (Afifi and Metts, 1998). Sometimes, the violation is considered minor when expected by individuals or when it has low importance. However, some expectancy violations can be major and thus substantially affect patient satisfaction and behavioral change.

Despite the prevalence of EVT in interpersonal communication, few studies have adopted EVT into the medical service domain (Bachman and Guerrero, 2006). Expectancy, with reference to PPC, is the anticipation or the belief about what is to be encountered in a medical consultation (Dean et al., 2019). Patient expectancy, which was historically overlooked during the medical consultation process, has gained increasing attention. In traditional medical encounters, physicians dominate the medical treatment process and the patient assumes a passive and subordinate role (Jiang, 2020). Physicians tend to only be interested in issues they consider important for their medical decision and ignore other patient feelings.

However, the focus on patient expectancy has continuously intensified in recent years with increasing attention to patient satisfaction and PCC (Lateef, 2011; Verlinde et al., 2012). The expectation of PCC refers to the patients’ hope to have their concerns, preferences, and questions heard and their needs effectively addressed. Patients increasingly expect a supportive context to participate more actively and play a central role in their health and healthcare. Patients are in need of affective empathy, self-participation, and to be included in the medical decision-making (Swaminath, 2007). In addition, some patients have expectation for control in the patient–physician interaction, such that the patients ask questions and state concerns and the physician is encouraging and supportive.

However, patient expectation is often not satisfied. In real life, the expectancy embodied in patient perception has often been negatively violated and thus influence patient satisfaction. The expectancy violation of PCC occurs if the patients’ perceptions of the extent to which healthcare providers adopt PCC to communicate is lower than their expectation (Moyer and Katz, 2007). The expectancy violation of PCC depends on several factors, including the characteristics of the communicator, context, and relationship (Burgoon, 1993). Many patients complained that the duration of medical consultation is inadequate and the consultation is hurried because the expectation of patients’ needs for information and emotional support is neglected (Swaminath, 2007). In addition, the usage of language, such as using a highly controlling or non-controlling language, may cause the expectancy violation of PCC in language expression (Averbeck, 2015). Patients’ negative expectancy violation negatively impacts their perceptions, communication, and behaviors in different healthcare encounters (Dean et al., 2019).

### OPPC Versus Offline Patient–Provider Communication in Patient Satisfaction and Expectation of PCC

One approach of examining patient satisfaction to OPPC is to compare OPPC with offline medical encounters. Whether OPPC would lead to as much patient satisfaction as offline patient–physician communication is an intriguing question. Patients’ visits to physicians, regardless of online or offline format, are driven by similar goals, that is, to be treated or cured. The differences between OPPC and offline medical encounters lie in the contextual variations between face-to-face and computer-mediated communication (Moyer and Katz, 2007).

Based on cues-filtered-out theories, computer-mediated communication, especially in the text format, can convey limited non-verbal cues and therefore is unable to fulfill social functions and restricts relationship development (Walther, 2011). The absence of non-verbal cues, such as eye contact and facial expressions, may inhibit the interaction and cause misunderstanding. In line with Short et al.'s (1976) findings, the fewer non-verbal cues a media channel transmit, the less warmth and involvement users experience. Therefore, OPPC is often considered less empathic and engaging than offline physician–patient communication because it often conveys fewer cues. Patients can feel verbal tones, observe facial expression, and interact with physicians through gestures in offline medical encounters (Hojat et al., 2017).

All these subtle behaviors can provide a sense of comfort and control for patients to increase their satisfaction. However, the absence of these cues in OPPC can also make people easily perceive the medical consultation as task-oriented and less satisfying (Moyer and Katz, 2007). In addition, OPPC is often asynchronous with text exchange, and the interaction can be interrupted by delay either from the physician’s or patient’s side (Deshpande et al., 2009). The lack of urgency reduces the approachability of each other, hinders positive communication and continued discussions between patient and physicians, and thus leads to low patient satisfaction (Lee and Zuercher, 2017). Thus, patient satisfaction is likely to be lower in the OPPC scenario compared with offline medical encounters.

Meanwhile, the expectancy violations of PCC tend to be intensified in the OPPC scenario as cues are filtered out. Achieving their expectation of affective need is difficult because patients could not receive meaningful cues from physicians (i.e., eye contact and facial expressions; Gorawara-Bhat and Cook, 2011). In addition, the asynchronicity of OPPC can cause a strong feeling of expectation violation regarding the swiftness of response and can considerably influence patients’ experience with physicians, especially when the patients are anxious to further understand their illness or medical situation (Moyer and Katz, 2007; Lee and Zuercher, 2017). Therefore, the expectancy violation of offline medical encounters in the OPPC tends to be high. We thus hypothesize the following:

*H1*: Patient satisfaction is lower in OPPC scenarios compared with offline medical encounters.

*H2*: Patient expectancy violation of PCC is higher in OPPC scenarios compared with offline medical encounters.

### Frequency of OPPC Usage

Patients may encounter various technological difficulties when using OPPC services (Robinson et al., 2013). From an evolving perspective, an adaptive process is likely to occur for the novel service, such that OPPC users may get accustomed to the limited cues to be received in the OPPC scenario as time passes (Rogers, 2015). Patients are likely to accept the cues available for interaction, consider the ease of use, and perceive the technology as reliable with frequent usage (Shahrabani and Mizrachi, 2016). Patients’ compliance with OPPC use can be associated with increased satisfaction.

Meanwhile, increased experiences with OPPC can lead to a more balanced perception to this healthcare format among patients (Jiang, 2020), which leads to lower expectancy violation. Patients with limited OPPC usage experience are likely to suffer from great expectancy violation of PCC, considering that the physicians are not as caring and empathetic as expected (Dean et al., 2019). Patients can better understand the technology restrictions, yield appropriate expectations, and suffer lesser expectancy violation of PCC after repeated usage of OPPC (Santana et al., 2010). Furthermore, OPPC is situated in the customer-centered culture of the wide online consumption atmosphere, and online patient rating systems serve as a driving factor for physicians to adopt a PCC approach and intentionally meet patient satisfaction (Corpman, 2013). The relationship between the frequency of OPPC and patient satisfaction may be mediated by the expectancy violation of PCC. We therefore propose the following hypotheses:

*H3*: Patients’ frequency of using OPPC is positively associated with their satisfaction toward OPPC.

*H4*: Expectancy violation of PCC mediates the relationship between patients’ frequency of PPC usage and their satisfaction in OPPC scenarios.

## Materials and Methods

A cross-sectional online survey was conducted from October 25–29, 2019 in China. China has one of the largest online medical service markets worldwide (Kantar Consulting, 2019). Approximately 190 million Chinese have consumed more than 310 million times of online medical consultation as of 2019. Online medical consultations in China can be performed through different local mobile apps and websites, such as Dingxiang Forum and Chunyu Doctor.

Multiple channels were used to distribute the survey links, including the app platform of a leading online medical service provider (“Health 160” app), its official WeChat account, and other online communities (see Diagram 1 for details). Health 160 app is based in Shenzhen, China but is available for medical services in over 200 Chinese cities. The accumulated number of online medical services reached 498 million person times (Health 160, 2020). In conjunction with the app, two rounds of system notifications were sent to 20,000 users who used online medical consultation services within the past month. The social media WeChat account of Health 160 also posted the participant recruitment poster with a link to the questionnaire.

Meanwhile, the survey link was also disseminated through online communities to solicit the early adopters of OPPC. According to Kantar Consulting’s, 2019 white paper on Internet Plus Medicine, most online medical consultation users were between the ages of 24 and 30 years old, especially young women in metropolitan cities (Kantar Consulting, 2019). New mothers are highly active users for OPPC. Thus, maternal–infant-themed online communities were used as seed groups for snowball sampling. Participants were encouraged to forward and share the questionnaire with others who might have conducted OPPC. Eligible participants were required to be over 18 years old, have experienced an online medical consultation once, and have experienced an offline medical consultation in the past 12 months. Each participant received 10 RMB as a reward and a chance to win other prizes through a lottery.

**Figure 1:**
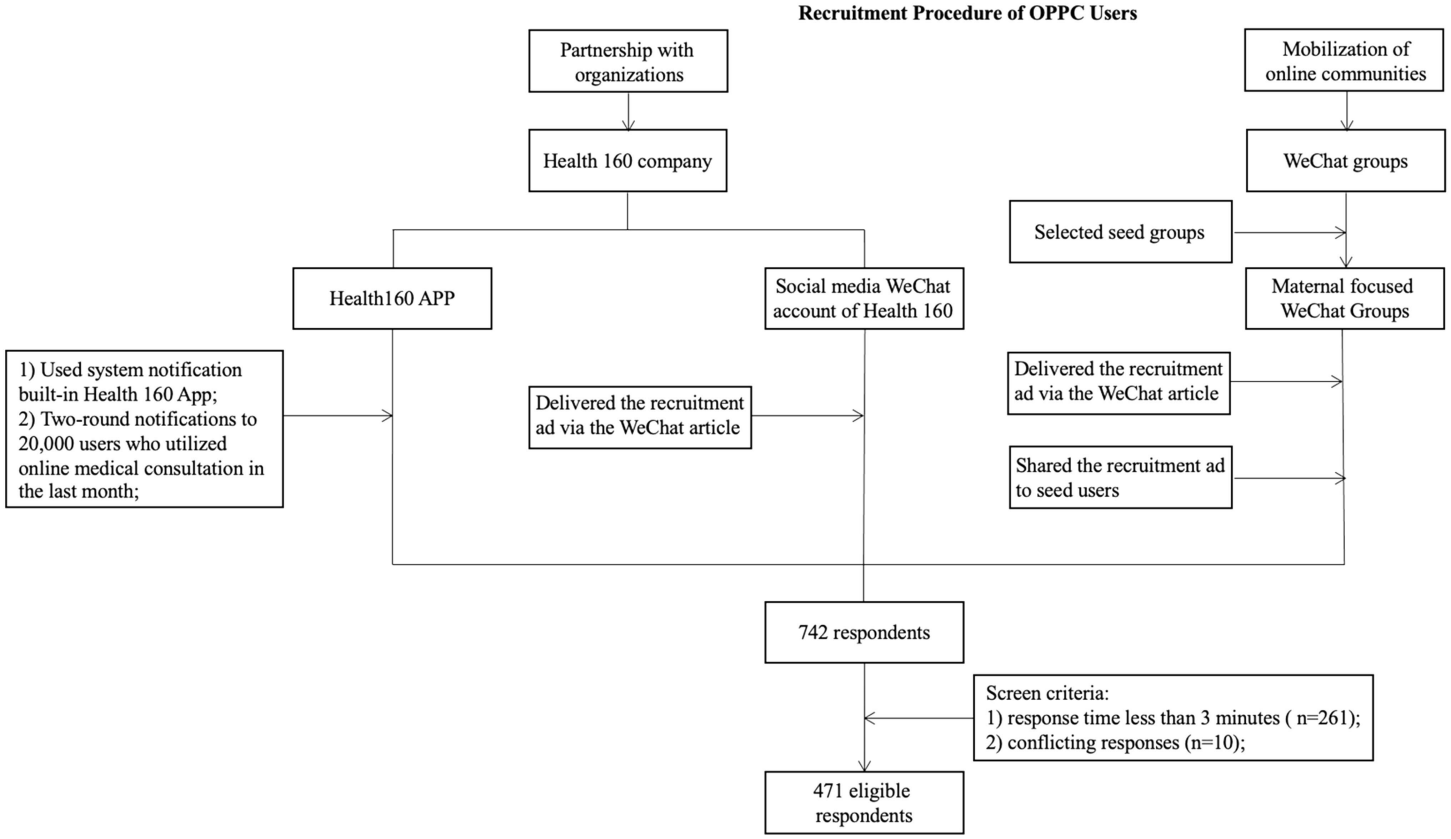
Participant recruitment process.

For quality control, 261 responses that were completed in less than 3 min and 10 responses that contained conflicting information were excluded. A total of 471 qualified responses were included in the final dataset (Figure 1).

### Measurement

The survey questionnaire was divided into two sections to collect the participants’ specific experiences in the scenarios of OPPC and offline medical encounters. In each section, similar questions were asked regarding usage frequency, patient satisfaction, and expectancy violations in the two scenarios.

#### Patient Satisfaction

Patient satisfaction refers to the evaluation of whether patients are satisfied with the healthcare process and outcomes. Patient satisfaction of OPPC and offline PPC were measured through the participants’ rating of the overall satisfaction with the quality of OPPC and offline PPC over the past 12 months. A five-point Likert scale ranging from 1(“strongly unsatisfied”) to 5 (“strongly satisfied”) was used.

#### Frequency of OPPC/Offline Medical Encounter

The frequency of OPPC/offline medical encounter was first measured through the participants’ self-reported frequencies of experiencing OPPC and offline PPC in the past 12 months. Participants were asked to fill in a specific number. Participants with only 1 to 2 times of OPPC usage were then grouped as having low usage frequency, those with 3 to 4 times of usage were categorized as having medium usage frequency, and those who used OPPC services at least 5 times were classified as having high usage frequency. The frequency of OPPC/offline medical encounter was recoded as an ordinal variable from 1 (low frequency of usage) to 3 (high frequency of usage) for analysis.

#### Expectancy Violation of PCC in OPPC/Offline Medical Encounter

Expectancy violation in this study refers to the gap between patients’ subjective expectation and their actual perception toward PCC. Instead of asking patients to directly report expectancy violation, this study measured the expectancy violation by the differences between the participants’ experienced PCC minus the expected PCC. The measurement of PCC was derived from a simplified PCC scale and patient-perceived patient-centeredness scale (Stewart et al., 2000). The scales were translated into Chinese for contextualization, and several rounds of primary interviews and pilot tests within the target population were conducted. The scales in Chinese were then modified on the basis of the feedback collected. The final PCC scales comprised five dimensions with 10 questions, including “fostering benign relationships,” “gathering sufficient information from patients,” “providing adequate information to patients,” “making shared decision,” and “guaranteeing communication effectiveness.” Each item was measured using a 5-point Likert scale ranging from 1 (“strongly disagree”) to 5 (“strongly agree”). The results of the reliability and exploratory factor analyses of the relevant scales indicate that the reliability and construct validity of the scale are acceptable (Supplementary Table 1). After calculation, the final score of expectancy violations varied from −4 to 4.

Expectation violation of PCC = expected PCC − experienced PCC.

#### Demographic Variables

Age (continuous variable), gender (female = 0, male = 1), monthly income (from 1 = lower than 3,000 RMB to 4 = higher than 8,000 RMB), health status (from 1 = basically healthy to 4 = with severe illness), and number of children (from 0 = no child to 2 = no less than 2 children) were assessed and were considered covariates in the multivariate statistical analysis.

### Statistics Analysis

Paired *t*-test was first used to test different research hypotheses and compare the patients’ expectations and perception of expectancy violation in OPPC and offline medical encounters. In addition, hierarchical multiple regression model was employed to explore how the frequencies of OPPC usage influence patient satisfaction. The PROCESS add-in function was utilized for mediation analysis (Hayes, 2009) and calculate the 95% confidence interval (CI) of the mediating effect and effect size. The bias-corrected non-parametric percentile bootstrap procedure with a sample size of 5,000 was employed to examine the mediation effect in this study. Age, gender, monthly income, health status, and number of children were the controlled variables.

## Results

### Participant Characteristics

More than half of the 471 participants were female (*n* = 281, 59.7%) and aged between 18 and 30 years old (*n* = 290, 61.6%). Nearly half of the participants earned more than 5,000 CNY per month (approximately 708 dollars; *n* = 212, 45%). Over one-fifth of the participants suffered from common diseases, long-time chronic diseases, or critical illnesses (*n* = 95, 20.2%). More than one-third (*n* = 198, 42%) of all respondents were parents of one or more children. The specific demographic characteristics are shown in Supplementary Table 1. More than half of the participants had low frequency of OPPC usage (*n* = 275, 58.4%), one-quarter of the participants had medium frequency of OPPC usage (*n* = 119, 25.3%), and 16.3% (*n* = 77) of the participants had high frequency of OPPC usage (Table 1).

**Table 1:** Demographic characteristics of participants (*n* = 471).

Variable	*n*	%
Gender		
Female	281	59.7
Male	190	40.3
Age		
18–30 years old	290	61.6
31 years old and above	181	38.4
Monthly income		
Less than 3,000 CNY (425 USD)	161	34.2
Between 3,001 and 5,000 CNY (708 USD)	98	20.8
Between 5,001 and 8,000 RMB (1,132 USD)	102	21.7
More than 8,000 RMB	110	23.3
Health status		
Basically healthy	376	79.8
Often suffer from common diseases	67	14.2
Have chronic diseases	26	5.5
Have other critical illnesses	2	0.4
Number of children		
Have no child	273	58.0
Have one child	140	29.7
Have two or more children	58	12.3

### Comparison Between OPPC and Offline Patient–Physician Communication

H1 proposes that patient satisfaction is lower in OPPC scenarios compared with offline medical encounters. The results of the paired *t*-test suggest that patient satisfaction in OPPC scenarios (*M* = 3.63, SD = 0.81) was significantly lower, compared with offline medical encounters (*M* = 3.75, SD = 0.80), *t*(470) = −3.33, *p* < 0.01. Thus, H1 was supported.

H2 proposes that patients’ expectancy violation of PCC is higher in OPPC scenarios compared with offline medical encounters. Based on the descriptive statistics (see Table 2), most of the respondents highly anticipated physicians to adopt PCC in OPPC (*M* = 4.13, SD = 0.75) and offline medical encounters (*M* = 4.06, SD = 0.77). However, their actual perceptions of PCC were lower in the OPPC scenario and led to a higher degree of expectancy violation. Approximately 60% of the respondents experienced negative expectancy violation in OPPC, and the percentage in offline medical encounter was 43.9%. Positive expectancy violation accounted for 17.2% in OPPC and 15.9% in offline PPC. The participants also met their expectancy in some situations; thus, no violations occurred in these situations. The results of the paired *t*-test of the expectancy violations suggest that the expectancy violation of PCC in the OPPC scenarios (*M* = 0.45, SD = 0.76) was significantly higher than that in the offline PPC scenarios (*M* = 0.27, SD = 0.69), *t*(470) = 4.82, *p* < 0.001. Therefore, H2 was also supported.

**Table 2:** Results of the comparative analyses of OPPC and offline PPC.

	OPPC (M[SD])	Offline PPC (M[SD])	*t*	*df*	Value of *p*
Expected PCC	4.13[0.75]	4.06[0.77]	2.509	470	0.012^*^
Experienced PCC	3.68[0.75]	3.79[0.77]	−3.280	470	0.001^**^
Expectancy violation of PCC	0.45[0.76]	0.27[0.69]	4.822	470	0.000^***^
Patient satisfaction	3.63[0.81]	3.75[0.80]	−3.332	470	0.001^**^

* *p* < 0.05;

** *p* < 0.01;

*** *p* < 0.001.

### Usage Frequency of OPPC and Offline PPC

H3 proposes that the patients’ frequency of OPPC usage positively associates with their satisfaction in OPPC scenarios. The results of regression analysis showed that the frequency of OPPC usage was positively associated with patient satisfaction (*β* = 0.209, *p* < 0.001) after normalizing gender, age, monthly income, health status, and number of children (Model 2 in Table 3). Thus, H3 was supported.

**Table 3:** Hierarchical multiple regression analysis results in OPPC.

Variables	Expectancy violation in OPPC	Patient’s satisfaction in OPPC	
	Model 1	Model 2	Model 3
Independent variable			
Frequency of OPPC usage	−0.093^*^	0.209^***^	0.185^***^
Mediator			
Expectancy violation in OPPC			−0.265^***^
Control variables			
Sex	−0.125^**^	0.048	0.015
Age	−0.003	−0.087	−0.088
Monthly income	0.099^*^	−0.047	−0.020
Number of children	−0.100	0.272^***^	0.245^***^
Health status	0.034	0.034	0.043
*R* ^2^	0.034	0.096	0.164
Adjusted *R*^2^	0.022	0.084	0.152
*F* value (sig. level)	2.756^*^	8.228^***^	12.992^***^
Number of cases	471	471	471

*p < 0.05; **p < 0.01; ***p < 0.001.

H4 proposes that expectancy violation mediates the relationship between patients’ frequency of OPPC usage and their satisfaction. Three regression models were conducted to examine the mediation hypothesis. The core coefficients for mediation analysis were as follows.


c=c’+ab.

As tested above, the frequency of OPPC usage was positively associated with patients’ satisfaction and indicated the existence of a direct effect (*c* = 0.209, *p* < 0.001). In addition, Model 1 in Table 3 demonstrates that the frequency of OPPC usage was negatively associated with the expectancy violation of PCC (*β* = −0.093, *p* < 0.05). This result indicates that the more the patients use OPPC, the less likely will they suffer from expectancy violation (*a* = −0.093, *p* < 0.05).

**Figure 2:**
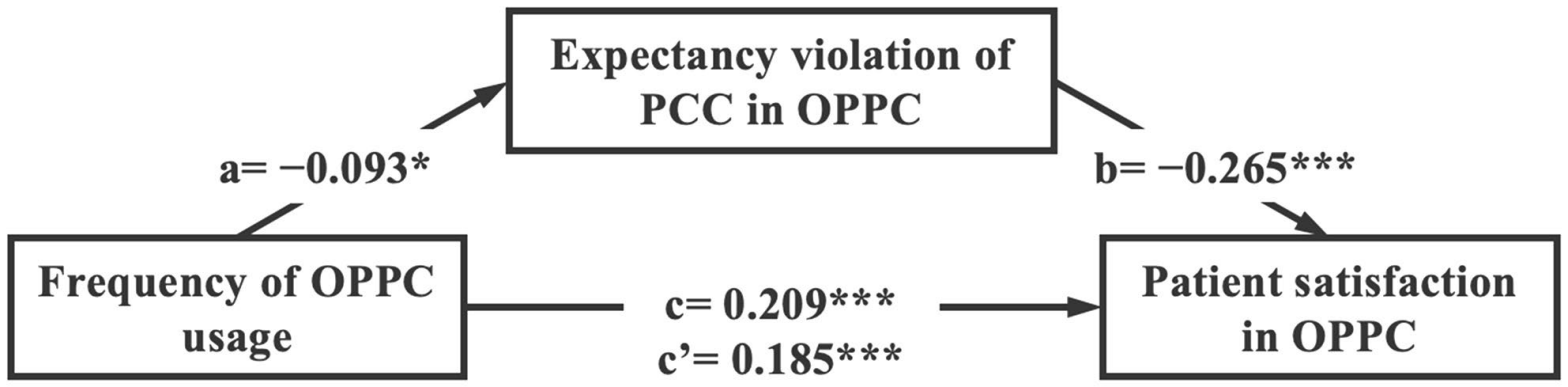
Standardized coefficients of mediation model. ^*^p < 0.05, ^**^p < 0.01, and ^***^p < 0.001.

Furthermore, Model 3 in Table 3 presents that the frequency of OPPC usage was positively associated with patient satisfaction (*β* = 0.185, *p* < 0.001), and the expectancy violation of PPC was negatively associated with patient satisfaction (*β* = −0.265, *p* < 0.001) when the frequency of OPPC usage and expectancy violation were introduced simultaneously into the model. These results indicate that more expectancy violation of PPC leads to lesser patient satisfaction (*b* = −0.265, *p* < 0.001), and the more frequent people use OPPC, the higher the patient satisfaction (*c’* = 0.185, *p* < 0.001). Figure 2 illustrates the results of the mediation analysis. The overall results revealed that *c’* was smaller than *c*, and all the conditions of the partial mediation were supported.

Bootstrapping analysis with 5,000 replicates was adopted to generate CIs and effect sizes to further evaluate the mediating effect of patients’ expectancy violation in OPPC. As indicated in Table 4, the expectancy violation of PPC was found to be a mediator in the relationship between the frequency of OPPC usage and patient satisfaction (95% CI = 0.0033–0.0542) when age, gender, monthly income, health status, and number of children were controlled. The direct effect accounted for 0.1949, and the indirect effect accounted for 0.0262 with a total effect size of 0.2211. The mediating effect size accounted for 11.85% of the total effect. Therefore, H4 was further supported.

**Table 4:** Mediating effect analysis results.

Model	Effect (SE)	95% CI	
		Lower	Upper
Total effect (Frequency of OPPC usage → Satisfaction)	0.2211^***^ (0.0474)	0.1278	0.3143
Direct effect (Frequency of OPPC usage → Satisfaction)	0.1949^***^ (0.0459)	0.1048	0.2851
Indirect effect (Frequency of OPPC usage → Expectancy violation → Satisfaction)	0.0262 (0.0130)	0.0033	0.0542

* *p* < 0.05; ^**^p < 0.01; ^*^p < 0.001.

## Discussion

The trend of transforming PPC from offline only to offline–online coexistence is desirable, but the approach to scale up the penetration of OPPC has not yet been clear (Jiang, 2019a). Users’ level of satisfaction with OPPC and how OPPC works must be understood to facilitate the wide adoption of OPPC (Gunter, 2005; Lee and Zuercher, 2017). The present study proposes a novel concept, that is, the expectancy violation of PCC, by intertwining EVT and PCC and verifying its mediating role between OPPC usage and patient satisfaction. This study is among the first few studies that extended the EVT into the medical context and is the first research to explicate expectancy with the PCC concept. The finding of this research can contribute to better understanding and improving the development of OPPC from patients’ perspective.

This study reveals that patient satisfaction toward OPPC is relatively lower compared with offline medical encounters. OPPC is relatively new and fast developing, especially after the COVID-19 pandemic outbreak when self-quarantine was widely encouraged and face-to-face medical consultation was prohibited (Boehm et al., 2020). The above-average score of patient satisfaction in the OPPC scenarios in this study is encouraging, considering that OPPC is in its early stage of development. However, patient satisfaction with OPPC was lower than offline PPC. This finding suggests that the development of OPPC still has a long way to go. Based on other propositions, the mature format of OPPC is yet to come (Gunter, 2005). Unsatisfaction toward OPPC is largely caused by the unavailability of sufficient cues during interaction (Silver, 2015; Dean et al., 2019) and the obstacles for health providers to offer accurate treatment advice without offsite observations (Lee and Zuercher, 2017). Concerns on the privacy, safety, and effectiveness of communication also hinder the wide acceptance of OPPC.

Some obstacles of OPPC, such as the absence of offsite observation to provide accurate treatments and diagnoses, can be solved to some extent with the development of wearable devices and medical-grade sensors in conjunction with cloud computing g(Lee and Zuercher, 2017). Patient satisfaction with OPPC will be promising by then. Prior studies have often considered OPPC as a substitute or a supplement to offline medical encounters, whereas recent studies suggested that OPPC and offline medical encounters are positively associated and complement each other (Jiang, 2020). This complementary relationship can be consolidated when OPPC accounts for a higher market share in healthcare delivery.

In addition, this study suggests that patients’ perception of PCC, particularly in meeting or violating the expectancy of PCC, is crucial for patient satisfaction in OPPC and offline medical encounters. Applying patient perception to evaluate the effectiveness of OPPC echoes to the trend of patient involvement in assessing consultation quality (Pawlikowska et al., 2010). This finding is aligned with prior studies, which found that patients’ perception of patient-centered behaviors strongly associates with patient satisfaction (Mallinger et al., 2005). Medical providers’ capability of providing PCC can be an indicator of their communication competence and can be highly related to patients’ expectancy satisfaction or violation (Kyrpychenko et al., 2021). Similar to the typical amplification effects, the greater the expectancy inserted in some pursuit, the greater the disappointment when unsatisfied (Miceli and Castelfranchi, 2014). This posit suggests that meeting patient expectation is highly demanded in OPPC and offline medical encounters. Therefore, managing the expectancy violation of PCC can be a future direction to guide patient–provider interaction, particularly to facilitate the development of OPPC.

This study reveals that the increase in the frequency of OPPC usage exerted a direct effect to the growth of patient satisfaction and the reduction of the expectancy violation of PCC, which further lead to higher patient satisfaction. These findings are consistent with the former research, which demonstrated the positive relationship between OPPC experience and patient satisfaction (Jiang, 2019a). These findings are also consistent with the diffusion of innovations theory in which an innovation is communicated over time among participants following a knowledge–persuasion–decision–implementation–conformation process (Rogers et al., 2019). Patients obtain knowledge about this new service and are exposed to its functions when they first use OPPC. After experiencing OPPC, the positive feedback can confirm patient decision, leading to further trials. In addition, based on the integrated spiral model of trust, confirmation of expectations from the interaction can reduce uncertainties, leading to an increased level of trust (Burgoon et al., 2021). Frequent usage of OPPC may signify patient’s trust toward OPPC practices. As patients’ favorable attitude toward OPPC proceeds to the second stage of persuasion, expectations tend to be more stable, and violations tend to be less frequent after repeated use of OPPC. Thus, patient satisfaction would also be higher. This process reveals a dynamic procedure of accepting a new model of medical services and an underlying negotiation about expectancy and experiences. It is a pity that this study examined the expectancy violation of PCC and did not measure patients’ pre-attitudes and impressions of doctors on an individual level, thus the effect of interpersonal impressions and relationships between patients and physicians during the online consultation process could not be examined. In line with the communicator reward valence concept under EVT theory, if the violator is regarded favorably—with what is called a high communicator reward valence—the violation may also be considered a positive act (Burgoon and Le Poire, 1993; Burgoon et al., 2021). Under this circumstance, patients’ expectations of OPPC being violated at some level may be turned into positive actions through online communication between them and their favorable doctors, increasing their preference for online consultation. Future study can consider the interpersonal relationship between patients and physicians and patients’ feelings toward the physician prior to online medical encounter when examining EVT online.

The findings of this study can provide insightful suggestions to expand online medical services. Previous studies have focused on training professionals to provide PCC in offline medical encounters (Shilling et al., 2003), but the approaches and strategies to offer PCC in the OPPC scenarios to meet patient expectation are underdeveloped. The quality of OPPC services is still concerning compared with offline medical encounters where services are quite systematic and definite (Al-Mahdi et al., 2015). Different from offline hospitals with structured training and education to provide PCC, online medical consultation platforms may lack specific training on PCC. The uniqueness of online scenarios where cues are filtered out and communication would be asynchronous forms natural barriers for the patients to perceive PCC. Therefore, extra efforts may be warranted to satisfy patients’ expectation of PCC. In addition, although OPPC has strived to enhance users’ experience by improving its designs (such as user-friendly user interface design and function design), increasing patients’ perceived PCC remains crucial for the development of OPPC. Prioritizing patients’ need to design OPPC platforms and provide services can capture more public interest toward OPPC and facilitate usage frequency (Gunter, 2005; Lee and Zuercher, 2017).

This study has obvious theoretical and practice implications. Theoretically, this study extended the patients’ perception of PCC to examine the development of OPPC, especially in providing a perspective on expectancy violation to explore the underlying mechanism of OPPC effectivity. The new concept of the expectancy violation of PCC is a remarkable negative mediator to patient satisfaction and deserves more attention to be attenuated for the expansion of OPPC. In practice, the development of OPPC is rapid but far from widely expanding because of the high threshold of medical services. Whether OPPC should be more open to embrace the opportunities of technological development to expand medical services or be more conservative to restrictively constrain its usage to ensure treatment efficacy remains a debate (Cao et al., 2018). The present study provides evidentiary support for further integrating new technologies with medical services. Efforts can be made to increase patients’ perception of PCC and reduce the expectancy violation of PCC during online medical consultation.

This study is not without limitations. First, this study was a cross-sectional survey; thus, findings on causality should be understood with caution. Second, this study did not use a representative sampling method because of the relatively low penetration rate of OPPC. Nevertheless, the investigation among early adopters of OPPC can satisfy the aim of this study to understand the underlying mechanisms of patient satisfaction toward OPPC. Moreover, this study relied on self-report data to investigate patients’ expected and experienced PCC during medical encounters and did not directly examine their actual process of encounters with doctors. Future studies can consider examining the interactions between patients and providers in the online context. More studies are needed to duplicate the study or revisit the relationship when OPPC is more developed or becomes more advanced.

## Data Availability Statement

The raw data supporting the conclusions of this article will be made available by the authors, without undue reservation.

## Ethics Statement

Ethical review and approval were not required for the study on human participants in accordance with the local legislation and institutional requirements. Written informed consent for participation was not required for this study in accordance with the national legislation and the institutional requirements.

## Author Contributions

BC and BH conceived the study. BC and YW collected the data. DW helped with the data analysis. BC and DW drafted the manuscript. BH revised the manuscript. All authors contributed to the article and approved the submitted version.

## Funding

This work has been supported by Major Project of the NationalSocial Science Fund of China (Grant/Award Number: 19ZDA324) and National Science Fund of China (Project No. 7210041729).

## Conflict of Interest

The authors declare that the research was conducted in the absence of any commercial or financial relationships that could be construed as a potential conflict of interest.

## Publisher’s Note

All claims expressed in this article are solely those of the authors and do not necessarily represent those of their affiliated organizations, or those of the publisher, the editors and the reviewers. Any product that may be evaluated in this article, or claim that may be made by its manufacturer, is not guaranteed or endorsed by the publisher.
