# Supplementary material for: Patient Expectation in China: Exploring Patient Satisfaction in Online and Offline Patient–Provider Communication
Source: Front Psychol. 2022 Jun 10;13:888657. doi: 10.3389/fpsyg.2022.888657 (PMC9226754; doi:10.3389/fpsyg.2022.888657)
Supplement: Supplementary file 1 [file Table_1.docx]

**Supplementary Table 1**

**The reliability and construct validity test of independent variables**

| Scales | Items | Cronbach's α | KMO | Bartlett's sphericity test | | |
| --- | --- | --- | --- | --- | --- | --- |
|  |  |  |  | Approximate  Chi-Square | df | sig. |
| Online expected PCC | 10 | 0.956 | 0.944 | 4811.509 | 45 | 0.000 |
| Online experienced PCC | 10 | 0.957 | 0.953 | 4638.273 | 45 | 0.000 |
| Offline expected PCC | 10 | 0.974 | 0.954 | 6429.695 | 45 | 0.000 |
| Offline experienced PCC | 10 | 0.969 | 0.958 | 5583.319 | 45 | 0.000 |
